# APPROACHES TO INVESTIGATING LUCIDITY IN ADRD: A PRELIMINARY RESEARCH FRAMEWORK TO GUIDE OPERATIONAL HARMONIZATION

**DOI:** 10.1093/geroni/igac059.355

**Published:** 2022-12-20

**Authors:** Andrea Gilmore-Bykovskyi, Joan Griffin, Kimberly Mueller, Sam Parnia, Ann Kolanowski

**Affiliations:** University of Wisconsin-Madison, Madison, Wisconsin, United States; Mayo Clinic, Rochester, Minnesota, United States; University of Wisconsin-Madison, Madison, Wisconsin, United States; NYU Grossman School Of Medicine, New York, New York, United States; Penn State University, State College, Pennsylvania, United States

## Abstract

Episodes of lucidity (EL) in Alzheimer’s disease and Alzheimer’s disease related dementias (AD/ADRD), also termed “paradoxical lucidity,” have garnered increasing attention as an important area of research. Efforts to study lucidity suffer from a lack of clear definitional criteria, inconsistent conceptualization and diverse approaches to operationalizing features of these events. To advance systematic investigation of EL in AD/ADRD, there is a need for clarity and precision in labeling event attributes, markers, and specific measurement strategies that enable operational harmonization across distinct approaches to investigating the relatively broad and nascent phenomenon. To that end, we propose a preliminary research framework to guide harmonization of approaches to investigating EL in AD/ADRD. Our goal is to provide a systematized approach to characterizing operational decisions that facilitates comparability across different methodological approaches and measurement strategies while allowing for exploration of multiple definitional criteria, measurement standards, and interpretive approaches.

